# Susac syndrome: about two cases

**DOI:** 10.11604/pamj.2019.33.145.17954

**Published:** 2019-06-26

**Authors:** Fatoumata Ba, El Hadji Makhtar Ba, Modou Mbacké Guèye, Marième Soda Diop Sène, Massar Diagne

**Affiliations:** 1 Health Sciences Unit, University Gaston Berger, Saint-Louis, Senegal; 2 Neurology Unit, Fann National Teaching Hospital, Saint-Louis, Senegal

**Keywords:** Susac syndrome, encephalopathy, cochleo-vestibular neuropathy, neurophysiology, Senegal

## Abstract

Susac syndrome is an autoimmune endothelopathy that affects precapillary arterioles of the brain, retina and inner ear. We report for the first time observations of two patients with Susac syndrome in Senegal.

## Introduction

Susac's syndrome is an autoimmune endothelopathy affecting precapillary arterioles of the brain, retina and inner ear [1]. This rare microangiopathy affects preferentially young women, 20-40 years old [2]. No risk factor has been described. The picture is manifested by a triad: encephalopathy, occlusions of the branches of the retinal artery and hypoacusis. But the triad is often incomplete at first, makes diagnosis difficult. It is a neurological pathology unknown and under diagnosed in our clinical practice. Until now, to our knowledge, no case of Susac syndrome has been published in Senegal. We report two clinical observations of strong suspicion of Susac syndrome.

## Patient and observation

**Case N°1**: a 49-year-old man with a history of recurrent bronchitis and profound deafness occurred at the age of 30, was admitted to the Neurology Unit of the Fann National Teaching Hospital for complex epilepsy drug-resistant. The anamnesis showed that the disorders started from infancy, by frequent crises: 1) sometimes generalized from the outset; 2) sometimes hemi-corporeal at the beginning then secondarily generalized; 3) sometimes complex partial with verbalization; 4) sometimes in the form of dreaming confusion or absences. A therapeutic dose of Phenobarbital, Carbamazepine, Keppra and Depakine, was successively given to the patient. For six months, the intensity, duration and frequency of seizures have worsened. Seizures were associated with temporo-spatial disorientation, attention and memory deficit. The neurological examination concluded to deafness, tremor attitude and bradypsychia. The remainder of the exam was entirely normal, as were the ocular examination, the biological and the hydroelectrolytic balance. The electroencephalography, done twice, at one-year interval, showed a bifronto-temporal irritative focus. Cerebral MRI, also done twice, at one-year interval, did not detect any abnormality. The recording of early auditory evoked potentials showed bilateral cochleo-vestibular neuropathy with severe perception deafness predominant on the right. The objective auditory threshold was 50 decibels (dB) on the left and 55 dB on the right. The audiogram showed a hypoacoustic perception predominant on low frequencies. The evolution has been marked by persistence of drug-resistant seizures, which have become more and more frequent. This required a transfer to Morocco to assess the indication of epilepsy surgery and/or the establishment of a neurostimulator.

**Case N°2**: a 43-year-old woman, sickle cell AS, who had a history of two comitial seizures untreated two years ago, was referred to the neurology unit for complete bilateral perceptual deafness, sudden onset. History has shown that the disorders began a few weeks ago with the brutal onset of complete deafness in conversation. The audiogram performed revealed severe bilateral deafness predominating on the right. The clinical examination of this patient was entirely normal. The fundus oculi were normal, as the biological assessment and hydroelectrolytic balance. Early auditory evoked potentials (PEAP) recording showed severe bilateral axono myelin cochleo-vestibular neuropathy predominantly right-sided with bilateral perceptual deafness predominating in the right side. The objective auditory threshold was 50 dB on the left and 60 dB on the right. Cerebral Magnetic resonance imaging (MRI) found uncollected bilateral oto-mastoiditis without clinical symptoms. The patient was treated with corticosteroid, antibiotic and anti-inflammatory drugs. Despite this treatment, no significant improvement in the clinical picture was observed. However, the oto-rhino-laryngologist considered cochlear implants and the patient was operated with good results. The seizures were not repeated and a control electroencephalogram (EEG) performed was normal.

## Discussion

Susac syndrome is an unknown and under diagnosed pathology because of the often-incomplete clinical presentation at the beginning and the lack of specificity of the encephalic involvement. Both patients have neurological signs such as partial or complex convulsive seizures, drug-resistant or spontaneous remission associated with complete hearing loss. Retinopathy was absent in our patients, contrasting with the data in the literature. Ocular involvement was very common. Thus, Dörr *et al.*, in their work, have shown that of the 304 cases published in recent years, 82% had exploitable data and among these 40% had ocular manifestations at the beginning of the disease and 97% during the course of the disease [3]. A complete eye examination should then be performed in the presence of any suspicion of Susac syndrome and repeated if necessary. Finding an occlusion of the branches of the retinal arteries, white retinal ischemia, the presence of soft nodules or cherry red spots were essential for diagnosis. MRI was normal in our patients, and in most cases reported, it was quite evocative with white matter involvement and brain micro-infarction. However, the delay to realize MRI was important, as abnormalities might appear during the course of the disease. Audiogram revealed severe bilateral deafness, according to the literature [4]. Electroencephalogram was often disturbed by Susac syndrome with encephalopathy, revealing diffuse brain suffering [5]. Therefore, the contributory nature of EEG to the diagnosis of Susac syndrome could be discussed. Patients were put on antiepileptic treatment (Case 1) and corticotherapy (Case 2) with antibiotics. This treatment differed from that proposed by Susac *et al.* [6], which might justify the fact that evolution was not favourable. Susac et al.’s protocol could be used as a test treatment, even in the absence of confirmation of diagnosis. Our limitation for reported cases was the lack of availability of retinal angiography.

## Conclusion

Susac syndrome affects all ethnic groups indiscriminately, although a high prevalence of the disease was noted in Caucasians. Because of the often-incomplete clinical picture, it is necessary to practice systematically a bottom of eye, a retinal angiography, an audiogram and an MRI in front of the slightest sign of appeal. We suggest conducting other prospective and multi-center studies to determine the prevalence of the disease in Senegal, to give the socio-demographic characteristics of the affected population, the evolutionary modalities and to evaluate the therapeutic possibilities.

## Competing interests

The authors declare no competing interests.
